# Rapid emergence of a virulent PB2 E627K variant during adaptation of highly pathogenic avian influenza H7N7 virus to mice

**DOI:** 10.1186/1743-422X-10-276

**Published:** 2013-09-05

**Authors:** Rineke MC de Jong, Norbert Stockhofe-Zurwieden, Eline S Verheij, Els A de Boer-Luijtze, Saskia JM Ruiter, Olav S de Leeuw, Lisette AHM Cornelissen

**Affiliations:** 1 Central Veterinary Institute of Wageningen UR, Lelystad, Netherlands

**Keywords:** Avian influenza virus, Ferret, H7N7, Mouse-adaptation, Pathogenicity, PB2 E627K

## Abstract

**Background:**

Highly pathogenic avian influenza (HPAI) viruses pose a potential human health threat as they can be transmitted directly from infected poultry to humans. During a large outbreak of HPAI H7N7 virus among poultry in The Netherlands in 2003, bird to human transmission was confirmed in 89 cases, of which one had a fatal outcome.

**Methods:**

To identify genetic determinants of virulence in a mammalian host, we passaged an avian H7N7/03 outbreak isolate in mouse lungs and evaluated the phenotype of the mouse-adapted variant in animal models and *in vitro*.

**Results:**

Three passages in mouse lungs were sufficient to select a variant that was highly virulent in mice. The virus had a MLD_50_ that was >4.3 logs lower than that of its non-lethal parental virus. Sequence analysis revealed a single mutation at position 627 in PB2, where the glutamic acid was changed to a lysine (E627K). The mouse-adapted virus has this mutation in common with the fatal human case isolate. The virus remained highly pathogenic for chickens after its passage in mice. In ferrets, the mouse-adapted virus induced more severe disease, replicated to higher titers in the lower respiratory tract and spread more efficiently to systemic organs compared with the parental virus. *In vitro*, the PB2 E627K mutation had a promoting effect on virus propagation in mammalian, but not in avian cells.

**Conclusions:**

Our results show that the E627K mutation in PB2 alone can be sufficient to convert an HPAI H7N7 virus of low virulence to a variant causing severe disease in mice and ferrets. The rapid emergence of the PB2 E627K mutant during mouse adaptation and its pathogenicity in ferrets emphasize the potential risk of HPAI H7N7 viruses for human health.

## Background

Highly pathogenic avian influenza (HPAI) viruses are the cause of a highly contagious and often fatal disease of poultry. Their pathogenicity is determined by the presence of multiple basic amino acids at the cleavage site of the HA protein, which renders HA susceptible to cleavage by furin-like host proteases [1-3]. The HA of avian influenza viruses mediates attachment of the virus to the cell through interaction with α2,3-linked sialic acid (SIA) receptors [4,5]. Avian influenza viruses do not usually infect humans, because epithelial cells in the upper respiratory tract of humans predominantly carry SIAs with α2,6 linkages [6]. During the epidemic caused by HPAI H5N1 viruses on the Asian continent, it became evident that these viruses could be transmitted directly from bird to human despite their preference for avian-type a2,3 SIA receptors [7]. In addition to HA, other viral proteins, notably polymerase proteins, are now recognized as determinants of host range and virulence of HPAI viruses in mammalian species (reviewed in [8]).

In 2003, the poultry industry in The Netherlands was hit by an outbreak of HPAI H7N7 virus, leading to the death and culling of more than 30 million birds [9]. Sero-epidemiological survey indicated that at least 89 persons had become infected by the time the epidemic ended [10]. Infected persons displayed signs of conjunctivitis and sometimes mild flu-like disease. One person became seriously ill and ultimately died from respiratory complications. Sequence analysis revealed 14 amino acid differences between the virus isolated from this fatal case (A/Netherlands/219/03) and the prototype chicken isolate (A/ch/Netherlands/1/03) [11]. One of those was present at position 627 of the PB2 protein, where the glutamic acid was substituted by a lysine (E627K). The PB2 E627K mutation distinguished the A/Netherlands/219/03 virus from other avian and human H7N7/03 outbreak isolates [12,13]. PB2 and the other viral polymerase proteins, PB1 and PA, form the polymerase complex of influenza viruses, which, along with NP, regulates replication and transcription of the viral genome [14,15]. Residue 627 of PB2 has been identified as an important determinant of host range and pathogenicity of avian influenza viruses in a mammalian host [16-20]. Studies in mice confirmed that this is also the case for H7N7/03 viruses. The A/Netherlands/219/03 virus was shown to be highly lethal to mice, in contrast to an H7N7/03 isolate obtained from a human conjunctivitis case (A/Netherlands/33/03). When 627K was introduced into PB2 of A/Netherlands/33/03, the virus acquired a lethal phenotype, whereas A/Netherlands/219/03 became low-virulent after introduction of glutamic acid at this position [21]. Pathogenicity studies with A/Netherlands/219/03 virus in ferrets also suggested an important role for PB2 627K, but a contribution of additional mutations in the genome was not excluded [22].

Although valuable data on virological aspects of H7N7/03 outbreak viruses have since become available [21,23], there is still little information on the molecular changes required for host adaptation and enhanced virulence of HPAI H7N7 viruses in mammals. Sequencing of avian viral isolates indicated that human adaptation markers evolved in poultry [12,23], but ‘the impact of these specific mutations for public health risk is yet uncertain’ [13]. Ferrets are considered suitable to predict the outcome of influenza virus infection in humans, because they have, unlike mice, predominantly human-type α2,6-linked SIA receptors in their upper respiratory tract [24-26]. Another advantage of this model is that ferrets exhibit clinical signs of influenza disease similar to humans [27]. The mouse can serve as a convenient mammalian model to study in a laboratory setting the molecular changes arising during adaptation of influenza viruses to a new host [24,28]. Serial lung passages in mice have been used to identify mutations that are critical for host change and enhanced virulence in mammals [29-31]. Such mouse adaptation experiments with H7N7/03 viruses have not been reported before. Here, we performed a mouse-adaptation experiment, in which one of the HPAI H7N7/03 chicken isolates, A/ch/Netherlands/621557/03, was serially passaged in mouse lungs. We then compared the virulence of the mouse-adapted variant with that of the parental chicken virus using the mouse and ferret as mammalian models. Our results show that three passages in mice were sufficient to select a PB2 E627K mutant that was highly lethal to mice and displayed enhanced pathogenicity in ferrets.

## Results

### Adaptation of chicken H7N7/03 virus to mice

Previously, we had observed that the A/ch/Netherlands/621557/03 isolate (designated as chH7N7 virus) was not lethal for mice, even at a dose of 10^6^ TCID_50_ (unpublished observations). To increase its virulence in mice (BALB/c), the chH7N7 virus was repeatedly passaged in mouse lungs. On day 4 of each passage, the lungs were harvested and virus titers determined in pooled lung homogenates. The initial infection with chH7N7 virus yielded a titer of 10^6.5^ TCID_50_/g tissue. Virus titers increased during subsequent passages to 10^8.0^ TCID_50_/g at the second passage (P2) and 10^8.5^ TCID_50_/g at the third passage (P3). Following inoculation of the P3 lung suspension, all 3 extra mice tested became ill and died 7 days after infection (data not shown).

### Virulence of mouse-adapted H7N7/03 virus in mice and chickens

**Figure 1:**
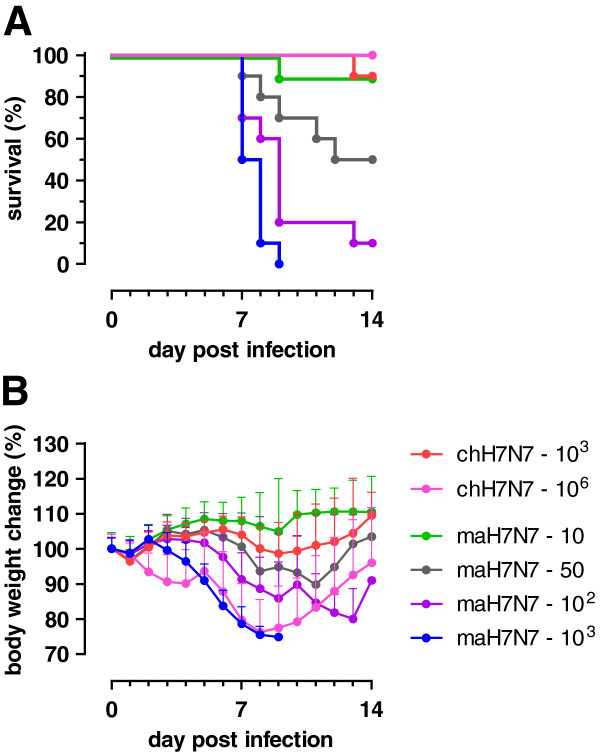
**Virulence of H7N7**/**03 viruses in mice.** Groups of 10 BALB/c mice were inoculated i.n. with maH7N7 or chH7N7 virus at the indicated dose (TCID_50_). Clinical signs and body weights were monitored daily until day 14 p.i. **A)** Kaplan-Meier survival curves, indicating the percentage of survivors over time. **B)** Body weight changes relative to the initial weight on day 0 (day of inoculation). Error bars indicate the standard deviations.

The virus present in pooled lung homogenate after 3 passages was propagated in embryonated chicken eggs to prepare the maH7N7 virus stock, which was subsequently used to assess the virulence of the mouse-adapted variant in mice. For this purpose, groups of 10 BALB/c mice were inoculated with increasing doses of maH7N7 virus. For comparison, two groups of 10 mice were inoculated with different doses of chH7N7 virus. The percentages of survival and mean body weights per group observed after infection are shown in Figure 1. Mice inoculated with 10^6^ TCID_50_ of chH7N7 virus showed considerable weight loss, but all survived. When a dose of 10^3^ TCID_50_ was given, one mouse died on day 13 post infection (p.i.), but all other mice survived without showing weight loss or other signs of disease. In contrast, mice inoculated with the same dose of maH7N7 virus began to lose weight soon after infection and all died 7 to 9 days later. Even with a dose as low as 50 TCID_50_ of maH7N7, 50% of the mice died. Thus, three mouse passages selected a virus with a MLD_50_ titer that was at least 4.3 logs lower than that of the chH7N7 virus (assuming that the MLD_50_ of the chH7N7 virus was higher than 10^6^ TCID_50_).

Based on the standard OIE test, avian influenza viruses with an intravenous pathogenicity index (IVPI) in 6-week-old chickens greater than 1.2 (out of a possible maximum of 3.0) are considered highly pathogenic. The chH7N7 virus had an IVPI of 2.9 [32]. We examined the pathogenicity of the maH7N7 virus in chickens following respiratory tract exposure by inoculating virus via the combined intranasal and intratracheal (IN/IT) route. The results indicated that the PB2 E627K mutant was lethal for chickens. Similar to what was previously observed after IN/IT administration of the chH7N7 virus [32], inoculation of maH7N7 virus resulted in rapid death of the birds; one chicken died on day 2 and the other 4 chickens died on day 3 p.i., corresponding with an IN/IT pathogenicity index of 2.6.

### Sequence analysis

We sequenced the entire genome of both viruses and found that the maH7N7 virus differed from the chH7N7 virus by only one amino acid at position 627 of PB2, where glutamic acid was changed into a lysine (E627K). Subsequent sequence analysis of the PB2 gene of virus present in P3 lung homogenates confirmed the presence of PB2 E627K. The PB2 sequence of virus from the P2 lung homogenate revealed that 627E was substituted already after the second mouse passage. Table 1 shows the amino acid sequence of the chicken virus selected for this study compared to that of other human and avian H7N7/03 viruses. The chH7N7 virus differed from the A/ch/Netherlands/1/03 prototype at only one position in NA (T164N). The maH7N7 and A/Netherlands/219/03 viruses had 14 amino acid differences, but shared the 627K in PB2.

**Table 1:** **Comparison of amino acid sequences of different H7N7**/**03 viruses**

	**Amino acid identity at indicated position**^***a***^															
**Virus**	**PB2**					**PA**	**HA**			**NA**					**NS**	
	**79**	**297**	**355**	**563**	**627**	**666**	**13**	**143**	**416**	**164**	**308**	**346**	**442**	**458**	**126**	**137**
A/ch/1/03	S	V	R	Q	E	F	I	A	K	T	N	A	T	P	K	V
chH7N7	.	.	.	.	.	.	.	.	.	N	.	.	.	.	.	.
maH7N7	.	.	.	.	**K**	.	.	.	.	N	.	.	.	.	.	.
A/33/03^*b*^	.	.	.	.	.	.	.	.	.	.	.	.	.	.	R	.
A/219/03^*c*^	I	I	K	R	**K**	L	S	T	R	.	S	V	A	S	.	I

^*a*^Amino acids are indicated by single letters; dots represent amino acids identical to the A/ch/Netherlands/1/03 (A/ch/1/03) prototype [11].

^*b*^Influenza A/Netherlands/33/03, isolated from human conjunctivitis case [11].

^*c*^Influenza A/Netherlands/219/03, isolated from fatal human case [11].

### Growth of H7N7/03 viruses in cell culture

**Figure 2:**
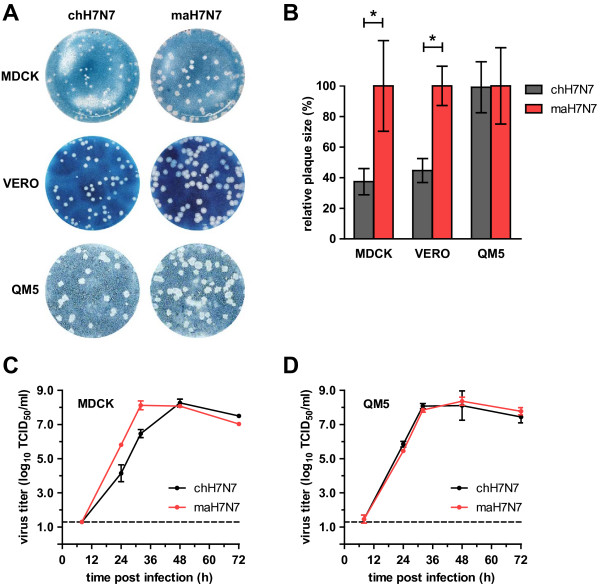
**Growth of H7N7/03 viruses in cell culture. A)** Plaque formation. MDCK, VERO or QM5 cells were infected with ~50 PFU of virus and stained with amido black after 7 days of incubation. **B)** Quantification of plaque size in MDCK, VERO and QM5 cells. The mean plaque size (measured in mm^2^) of chH7N7 is plotted relative to that of maH7N7 (set at 100%). Error bars represent the standard deviations. The asterisk indicates significant difference in mean plaque size (P < 0.05). **C**, **D)** Growth kinetics of H7N7/03 viruses. MDCK **(C)** or QM5 cells **(D)** were infected with chH7N7 or maH7N7 virus at an m.o.i. of 0.001 TCID_50_ per cell. Culture supernatants were harvested at 8, 24, 32, 48 and 72 h p.i. and virus titers were determined by end-point titration in MDCK cells. Shown are means from duplicate experiments with error bars indicating standard deviations. The dotted line indicates the lower limit of virus detection. Undetectable titers were assigned a value of 1.3.

*In vitro* growth of maH7N7 and its parental virus was assessed by analysis of plaque formation in cell cultures. As shown in Figure 2A and B, infection of MDCK cells with maH7N7 virus resulted in plaques that were 2.7-fold larger than those of the chH7N7 virus. The maH7N7 virus produced larger plaques (2.2-fold) also in monkey kidney-derived VERO cells. No difference in plaque size between the maH7N7 and chH7N7 viruses was observed in QM5 cells, a quail myogenic cell line [33]. When the replication kinetics of both viruses were examined, we found that the maH7N7 virus replicated faster than the chH7N7 virus in MDCK cells, yielding titers that were 1.6 logs higher at 24 and 32 h p.i. (Figure 2C). However, the maH7N7 and chH7N7 viruses produced similar growth curves in QM5 cells (Figure 2D), which was consistent with their plaque phenotypes. These results indicate that the maH7N7 virus was more efficient at replicating in mammalian cells than the chH7N7 virus.

### Pathogenicity of H7N7/03 viruses in ferrets

**Figure 3:**
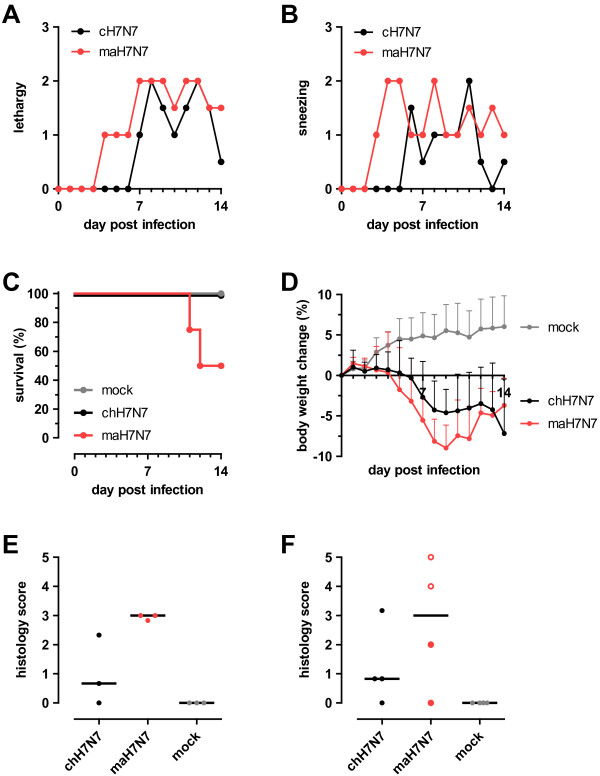
**Pathogenicity of H7N7/03 viruses in ferrets.** Ferrets were inoculated i.n. with 10^7.4^ TCID_50_ of either maH7N7 or chH7N7 virus. As negative control, one group of ferrets was mock-infected. On day 5 p.i., 3 of the 7 animals per group were euthanized for post mortem examination. The remaining animals were monitored for clinical signs and body weight changes during 14 days p.i. **A)** Median scores for lethargy. **B)** Median scores for sneezing (if a ferret was euthanized, the last assigned score was carried forward). **C)** Kaplan-Meier survival curves. **D)** Mean body weight changes relative to baseline weights at day 0. Only 2 ferrets of the maH7N7 group remained on day 13 p.i. Error bars represent standard deviations. **E**, **F)** Semi-quantitative scoring of histopathological changes in lung tissues. Microscopic lung changes were scored on a scale of 0 to 5 as detailed in the Methods section. Each point represents the histology score of an individual animal and horizontal bars represent the median group scores. Lungs were examined **E)** on day 5 and **F)** on day 14 p.i. or when an animal needed to be euthanized before the end of the study period (one on day 11 and 12 each, shown with open circles).

Next, we examined whether the PB2 E627K mutation in the chicken virus also had an effect on its pathogenicity in ferrets. To this end, two groups of 7 ferrets each were inoculated with 10^7.4^ TCID_50_ of maH7N7 or chH7N7 virus and one group was mock-infected. In each group, 3 animals were euthanized on day 5 p.i. for post mortem examination; the other 4 animals were monitored for clinical signs and body weight during 14 days after infection. From day 3 onwards, clinical signs were observed in maH7N7-infected ferrets, including lethargy, sneezing and respiratory distress. These symptoms were also observed in ferrets infected with chH7N7 virus, but were generally milder, had a later onset and the animals recovered earlier (Figure 3A and B). Furthermore, ferrets in the maH7N7-infected group displayed loss of appetite from day 6 to 9 p.i., whereas reduction of food intake was reported only on day 7 p.i. for the chH7N7-infected group. All chH7N7-infected ferrets survived (Figure 3C). Of the ferrets infected with maH7N7 virus, two needed to be euthanized (one on day 11 and 12 each) because of severe illness; one animal exhibited severe lethargy (a score of 3), weakness and trembling, while the other was persistently lethargic (a score of 2 for 8 days). Ferrets in the chH7N7 group lost maximal ~5% of their initial body weight, whereas those in the maH7N7 group lost maximal ~9% of their weight (Figure 3D). On day 14 p.i., one chH7N7-infected animal showed a sudden drop in body weight, but no severe signs of disease or pathological changes in the lungs. Mock-infected animals did not show any signs of disease and gained weight over time. All chH7N7 and maH7N7 virus-infected ferrets exhibited H7 hemagglutination inhibiting (HI) antibody titers (geometric mean titers of 420 and 680, respectively) on day 14 p.i.

Pathological examination of the lungs at day 5 p.i. revealed foci of consolidation in two chH7N7-infected ferrets in 1 or 2 lobes affecting ~1 or 3% of lung tissue (Table 2). Comparable lesions were observed in all three maH7N7-infected animals in 1 to 3 lobes affecting ~3 to 5% of lung tissue. Histopathological changes in the lungs were similar in character in both infection groups; however, the lesions were more severe and affected a greater percentage of tissue in lungs infected with maH7N7 virus than in lungs infected with chH7N7 virus (Figure 3E). The lungs of maH7N7-infected ferrets showed alveolar hemorrhages, infiltrations of granulocytes, lymphocytes and histiocytes in the alveoli, and necrotizing bronchiolitis with obliteration of bronchioli. No histopathological changes were found in other organs, except in one chH7N7-infected animal, which had foci of mononuclear cell infiltrates in the liver.

**Table 2:** **Gross pathology in lungs of ferrets infected with H7N7**/**03 virus**

	**Gross pathological lung lesions on indicated time p**.**i**.					
	**Day 5**			**End of the study**^***a***^		
**Virus**	**No. of animals**^***b***^	**No. of lobes**	**Area affected (%)**	**No. of animals**	**No. of lobes**	**Area affected (%)**
chH7N7	2/3	1, 2	1, 3	3/4	1, 1, 2	1, 3, 3
maH7N7	3/3	1, 2, 3	3, 3, 5	3/4	3, 5, 6	10, 25, 30
mock	0/3	x	x	0/4	x	x

^*a*^On day 11 and 12 for the two fatal cases, on day 14 for all survivors.

^*b*^Number of animals with gross lung pathology/total number of animals.

x = no pathological findings.

Gross pathology at the end of the study revealed that, in the lungs of three of four animals infected with chH7N7 virus, foci of consolidation were present in 1 or 2 lobes, involving ~1 to 3% of lung tissue (Table 2). Multifocal to coalescing consolidated areas were observed in 5 or 6 lobes in total affecting ~25 to 30% of the lungs of the two fatal cases of maH7N7 infection and in 3 lobes with ~10% lung tissue affected in one of the two surviving animals of this group. Histopathology of lungs of ferrets infected with chH7N7 virus revealed only a few, small areas with atelectasis or slight interstitial pneumonia, sometimes with mild, focal bronchitis. Lesions were more severe and extended to larger areas in the lungs of ferrets infected with maH7N7 virus (Figure 3F). In these lungs, we observed multifocal, interstitial pneumonia, alveolar hyperplasia, peribronchitis and, particularly in the two fatal cases, fibrinous alveolar exudate with many mononuclear cells. There was slight to moderate infiltration of lymphocytes and histiocytes in liver tissues from animals of both infection groups. Focal lymphohistiocytic encephalitis was seen in both fatal cases of maH7N7 infection. No pathological changes were detected in organs of the mock-infected controls. In conclusion, the maH7N7 virus generally induced more severe disease with more extensive lung pathology than did the chH7N7 virus.

### Replication of H7N7/03 viruses in ferrets

**Figure 4:**
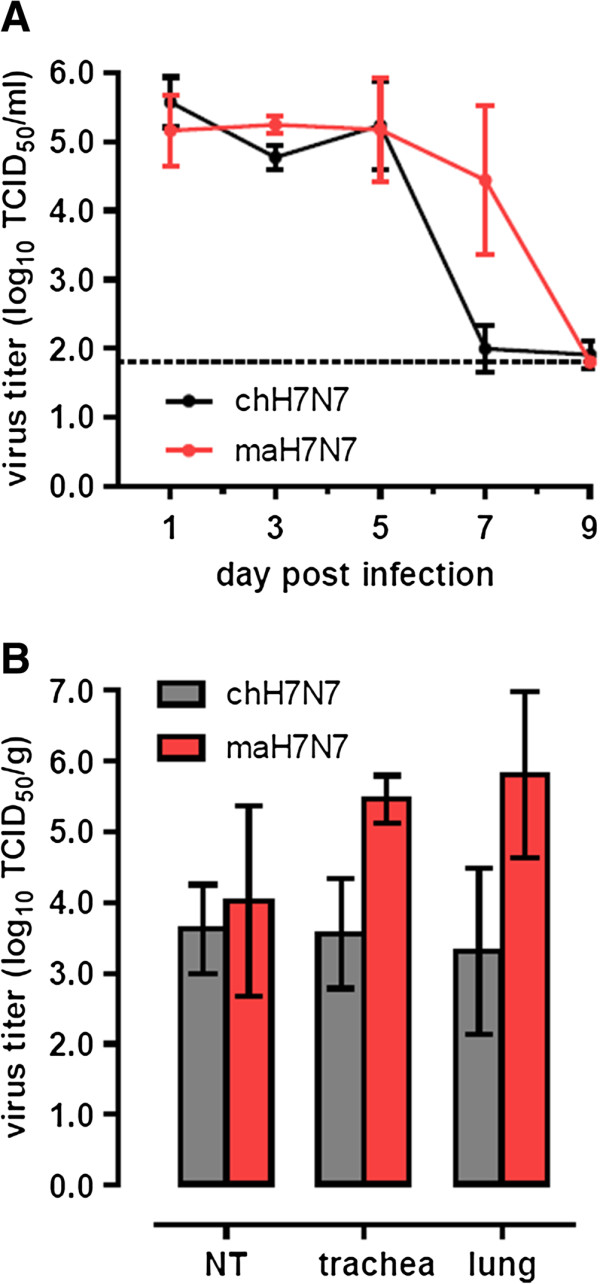
**Replication of H7N7/03 viruses in the upper and lower respiratory tract of ferrets. A)** Ferrets were i.n. inoculated with 10^7.4^ TCID_50_ of virus and nasal washes collected at 1, 3, 5, 7 and 9 days p.i. were titrated in MDCK cells. Mean virus titers (n=4) are shown with error bars indicating standard deviations. The dotted line indicates the lower limit of detection. **B)** Mean virus titers in nasal turbinate (NT), trachea and lung tissues collected from ferrets (n=3) on day 5 p.i. Error bars represent the standard deviations.

Nasal washes were collected from ferrets on alternate days from day 1 to 9 p.i. and tested for the presence of virus. The nasal wash titers of the maH7N7 and chH7N7 viruses were comparable until day 5 p.i. (range, 4.3 - 5.9 TCID_50_/ml; Figure 4A). The chH7N7 virus was no longer detected or shed only at low level by day 7 p.i. At this time point, maH7N7 virus continued being shed with a mean titer that was 2.4 logs higher than that of the chH7N7 virus (range, 3.3 - 5.8 TCID_50_/ml).

After necropsy on day 5 p.i., viral loads were determined in respiratory tissues and other organs. The results are shown in Figure 4B and Table 3. Virus was detected in all nasal turbinate samples with similar mean titers for both viruses, although only two samples could be tested in the chH7N7 group. Viral titers in the trachea and lungs of ferrets infected with maH7N7 virus tended to be higher (2 and 2.5 logs on average, respectively) than those measured after infection with chH7N7 virus. In two of three maH7N7-infected ferrets, virus was detected in multiple organs, including the intestines, kidney, spleen, liver, heart and brain, indicating systemic infection. Infectious chH7N7 virus was recovered only from the respiratory tract, except in one animal, which had a virus titer in the brain. The recovery of chH7N7 from the brain, and not from other extra-respiratory organs, of one animal is consistent with previous observations [21,22] and was suggested to be due to virus ascending via sensory nerves directly from the nose to the brain and not the result of systemic virus distribution [34,35]. Since olfactory bulb tissues were not sampled separately, this could not be confirmed.

**Table 3:** Virus recovery from organ tissues of ferrets

		**Virus titers in organs collected from ferrets on day 5 p**.**i**.^***a***^												
**Virus**	**Ferret**	**NT**^***b***^	**Tr**	**L1**^***c***^	**L2**	**L3**	**L4**	**Ileum**	**Jejun**	**Kidney**	**Liver**	**Spleen**	**Heart**	**Brain**
chH7N7	#1	4.1	4.3	2.4	5.0	2.4	2.6	−	−	−	−	−	−	−
	#2	nd	3.7	2.4	2.4	2.4	4.9	−	−	−	−	−	−	2.5
	#3	3.2	2.7	4.8	4.7	2.4	3.2	−	−	−	−	−	−	−
maH7N7	#1	5.6	5.1	3.8	4.4	5.2	4.0	−	−	−	−	−	−	−
	#2	3.4	5.7	6.3	6.3	6.3	6.8	3.0	3.4	−	2.5	−	2.7	3.0
	#3	3.2	5.7	6.2	7.0	7.4	5.9	2.7	−	2.5	−	2.6	3.0	−

^*a*^Virus titers expressed as log_10_ TCID_50_ per gram tissue.

^*b*^NT = nasal turbinate; Tr = trachea; Jejun = jejunum.

^*c*^Lung tissues were sampled from left cranial (L1), right cranial (L2), left caudal (L3) and right caudal (L4) lobes.

nd = not determined; − = <2.4 log_10_ TCID_50_/g.

## Discussion

The high incidence of human infections during the Dutch epidemic in 2003, including one fatal case, has raised concerns regarding the potential risk of HPAI H7N7 viruses to human health. To identify molecular changes that are critical for host adaptation and pathogenicity of these viruses in mammals, we generated a mouse-adapted variant of one of the avian H7N7/03 viruses isolated at the earliest stage of the 2003 outbreak and evaluated its phenotype in animal models and *in vitro*.

Mouse passaging rapidly gave rise to a variant that differed from its avian parent only by the PB2 E627K mutation. The mouse-adapted variant has this mutation in common with the A/Netherlands/219/03 virus, indicating that experimental adaptation of the H7N7/03 virus to mice selected the same mutation as occurred during adaptation to humans under natural conditions. The PB2 E627K mutant was significantly more virulent for mice than its non-lethal parental virus (> 4.3 logs decrease in MLD_50_ value). A dose of 10^3^ TCID_50_ resulted in 100% lethality, which was in agreement with the lethality observed for the A/Netherlands/219/03 virus [21,22]. Evaluation of both viruses in ferrets revealed that the maH7N7 virus generally caused more severe disease with more pronounced lung pathology, necessitating euthanasia of two of the four animals. Although mean viral titers in the upper respiratory tract were initially not markedly different, the mouse-adapted virus was shed for one day longer. The virus exhibited clear replicative advantage over the chH7N7 virus in the lower respiratory tract and spread more efficiently to systemic organs. The contribution of PB2 627K to pathogenicity of the A/Netherlands/219/03 virus has previously been demonstrated in mice [21]. Our results show that mouse adaptation of HPAI H7N7 virus is driven by PB2 and that PB2 E627K is an important virulence factor of the virus not only in mice, but also in ferrets.

Viral growth performances in cultured cells correlated with the pathogenicity or level of virus replication observed in the animal models. The maH7N7 virus showed growth characteristics comparable to that of the parental virus in cells of avian origin and retained intranasal pathogenicity in chickens; however, it grew with greater efficiency in MDCK (canine) and VERO (monkey) cells and replicated to higher titers in lungs of mice and ferrets. Others have already shown that, in agreement to observations for other influenza subtypes, PB2 627K in A/Netherlands/219/03 had a promoting effect on virus replication in mammalian cells by increasing the activity of the avian polymerase [23]. As the PB2 sequence of the maH7N7 virus is identical to that of A/Netherlands/219/03 (Table 1), we conclude that maH7N7 pathogenicity in the mouse and ferret models solely resulted from the increased potential of the adapted PB2 polymerase to replicate in cells from different mammalian species.

Previous work has shown that mutation A143T in HA induced enhanced replication and tissue distribution, but not pathogenicity, of the A/Netherlands/219/03 virus in mice. This mutation, adding a potential glycosylation site at position 141, was found responsible for a change in SIA receptor specificity from exclusively α2,3 to both α2,3 and α2,6 linkages [21,23]. Altered receptor binding properties of HA seemed not to be critical for virulence and systemic spread of the maH7N7 virus in ferrets, given the fact that the chicken virus used in our study binds only to α2,3-linked SIAs [23] and its adaptation to mice was not accompanied by changes in HA. In this regard, the maH7N7 virus shows similarity to the HPAI A/Vietnam/1203/04 H5N1 virus that also spread to different organs in ferrets despite having avian-type SIA specificity. It was suggested that reduced ability to attach to mammalian host cells can be compensated by more efficient replication of the genome by PB2 627K, resulting in higher yield of progeny virus in an infected cell [36].

De Wit and co-workers [23] used *in vitro* systems to investigate the molecular basis of differences in pathogenicity between the human A/Netherlands/219/03 and A/Netherlands/33/03 viruses. They found that, in addition to PB2 E627K and HA A143T, amino acid substitutions in PA (F666L) and NA (N308S, A346V, T442A and P458S; Table 1) accounted for enhanced replication of A/Netherlands/219/03 virus in human cells, suggesting a causal link to adaptation and fatal disease in the human host. Genetic analysis of a large panel of outbreak viruses isolated from poultry revealed that, with the exception of PB2 E627K, these amino acid substitutions were already present in poultry viruses [12,23] and obviously did not result from human adaptation. It is noteworthy that the chicken isolate used in our study did not have any of these substitutions or other known potential virulence/human adaptation markers [13,30]. Yet, the virus was able to establish a productive infection in ferrets, yielding substantial titers, while the PB2 E627K substitution appeared to be sufficient for induction of severe disease. These observations suggest that HPAI H7N7 viruses may have pathogenic potential in humans without involvement of additional, replication-enhancing amino acids, although some of them may facilitate host adaptation or, in combination with PB2 627K, further increase the virulence. It will be of interest to passage H7N7/03 viruses in ferrets to establish whether the PB2 E627K mutant emerges within a similar small number of passages when viruses are replicating in an human-like α2,6 SIA-rich environment.

## Conclusions

This study shows that the PB2 E627K mutation, also present in the fatal human case virus, can be sufficient to convert a HPAI H7N7 virus of low virulence into a variant causing severe disease and death in mice and ferrets. Our results underline the importance of PB2 627K in modulating host specificity and pathogenicity in mammals and emphasize the potential risk of HPAI H7N7 viruses for human health.

## Methods

### Cells and viruses

Madin-Darby canine kidney (MDCK) cells and VERO (African green monkey kidney) cells were cultured in Dulbecco minimal essential medium (DMEM) supplemented with 5% fetal bovine serum (FBS) and antibiotics. Quail myoblast (QM5) cells were cultured in QT35 complete medium (Life Technology) with the same supplements. The virus used in this study was A/ch/Netherlands/621557/03, an HPAI H7N7 virus isolated from chickens on the index farm of the outbreak in The Netherlands in 2003 [32]. The virus was propagated in embryonated chicken eggs to produce a virus stock (designated chH7N7). All experimental work with HPAI virus was performed under BSL3 conditions in the high-containment unit of Central Veterinary Institute, Lelystad.

### RNA isolation, RT-PCR amplification, and DNA sequencing

Viral RNA was isolated from allantoic fluid of infected chicken eggs using a High Pure Viral RNA kit (Roche) according to the manufacturer’s protocol. cDNA was synthesized using Superscript II (Invitrogen) and amplified by PCR using *Z*-*Taq* polymerase (Takara Bio Inc.) and primers matching the noncoding sequence of each gene segment. The PCR products were excised from an agarose gel and then purified using a Zymoclean kit (Zymo Research) as indicated by the manufacturer. The cDNA of maH7N7 was sequenced by direct dideoxy-terminated cycle sequencing using BigDye terminator v1.1 sequencing kit (Applied Biosystems) and a 3130 genetic analyzer sequencer (Applied Biosystems). cDNAs of chH7N7 virus were produced by PCR, isolated and cloned into the pGEM-T vector (Promega). Sequences were determined by sequencing at least two different clones obtained from independent PCR reactions. For sequence alignments the SeqMan II software (DNAstar) was used.

### Mouse experiments

Female, 7 to 9-week-old BALB/c mice were purchased from Charles River Laboratories. The chH7N7 virus was serially passaged by intranasal (i.n.) inoculation (50 μl) of groups of three mice, starting with a dose of 10^5^ TCID_50_. After 4 days, the mice were euthanized; their lungs were pooled and homogenized in PBS to produce a 20% (w/v) suspension. The clarified suspension was used as inoculum in the next passage using again three mice. After each passage, a sample of the lung suspension was titrated in MDCK cells. Three extra mice were inoculated with the passage 3 (P3) lung homogenate and maintained to monitor the course of disease. Virus present in the lung homogenate of P3 was propagated in embryonated chicken eggs to produce a virus stock (designated maH7N7) for use in further experiments.

In the next experiment, the 50% mouse lethal dose (MLD_50_) was determined by inoculating four groups of 10 BALB/c mice (female, 7 weeks of age) with increasing doses of maH7N7 virus. For comparison, two other groups of 10 mice were inoculated with different doses of chH7N7 virus. Survival and body weights were recorded daily during 14 days.

### Chicken experiment

The pathogenicity of maH7N7 was determined in 5 SPF white Leghorn chickens (Charles River Laboratories) of 6 weeks of age. These birds were inoculated with 0.2 ml of diluted allantoic fluid (0.1 ml intranasally [IN] and 0.1 ml intratracheally [IT]) containing 10^5^ TCID_50_ of virus and observed daily for clinical signs over a period of 10 days. The birds received a score of 0 if they appeared normal, 1 if they were ill, 2 if they were severely ill and 3 if they were dead. The IN/IT pathogenicity index was calculated as the mean score per bird per observation.

### Ferret experiment

For this experiment, 21 male ferrets of approximately one year of age were purchased from a local breeder. All ferrets had been tested seronegative for H7N7/03 influenza virus and circulating influenza A viruses, but possessed HI antibodies (ranging from 20 to 80) against circulating influenza B/Brisbane/60/2008 virus. After an acclimatization period of one week, groups of 7 ferrets were anaesthetized by intramuscular injection of medetomidine (0.1 mg/kg) and inoculated i.n. with 10^7.4^ TCID_50_ of virus (0.5 ml). As a negative control, one group received an equal volume of sterile allantoic fluid (mock infection). Five days later, three ferrets per group were sacrificed and examined for gross pathological lesions. Tissue samples were taken from the respiratory tract, lungs, visceral organs, heart and brain for histopathological and virological analyses. The remaining four animals were weighed daily and observed for signs of illness during 14 days p.i. Clinical signs were recorded using a scoring system ranging from 0 (absence of signs) to 3 (severe signs) for activity level [27], sneezing, respiratory distress, neurological signs, nasal/ocular discharge and diarrhea. To assess virus shedding, nasal washes were collected on day 1, 3, 5, 7 and 9 p.i. as previously described [37]. At the end of the study, all surviving animals were euthanized and bled. Sera were tested for the presence of antibodies by standard HI assay using 1% chicken red blood cells and 4 hemagglutinating units of the chH7N7 virus. Titers were expressed as the reciprocal of the highest serum dilution inhibiting agglutination. The animals were necropsied and the same tissue samples were taken as described above.

### Gross pathology and histopathological examination

The organs were examined macroscopically and lesions described. Gross lung lesions were recorded by indicating affected areas on a drawing representing all lung lobes. For histopathology, organ tissues were fixed in 10% neutral buffered formalin, embedded in paraffin and sectioned into 5 μm-slides. The sections were stained with hematoxylin-eosin and examined microscopically by a pathologist blinded to the treatment of the animals. The characteristics and degree of histopathological changes in the lungs were evaluated at 10x objective magnification in 4 to 6 slides per lung in 5 or 6 microscopic fields each and scored on a scale of 0 to 5 as follows: 0 = absence of changes; 1 = few inflammatory cells in <10% area in at least one microscopic field; 2–5 = alveolitis, bronchiolitis or (interstitial) pneumonia in <10%, 10-50%, 50-75% and 75-100% area, respectively, in at least one microscopic field.

### Virus titration

Nasal washes were clarified by low-speed centrifugation and the supernatants were stored at −70°C until analysis. Organ tissue samples were weighed, homogenized and diluted in phosphate buffered saline (PBS) or DMEM containing antibiotics to prepare 20% (w/v) suspensions. Clarified organ suspensions were either directly titrated or stored at −70°C before titrations were performed. Virus titers were determined by the end-point dilution method as described [38]. Briefly, 10-fold serial dilutions of the samples were added to MDCK cell cultures. After two or three days of incubation at 37°C, the cells were permeabilized, fixed in 4% paraformaldehyde and subjected to immunoperoxidase staining [39] using NP-specific monoclonal antibody HB65 (CVI, Lelystad). Virus titers were calculated according to Reed and Münch and expressed as 50% tissue culture infectious dose (TCID_50_).

### Plaque assay

Confluent cell monolayers grown in 6-well plates were washed twice with PBS followed by inoculation with ~50 plaque forming units (PFU) of virus. At 2 h p.i., the inoculum was removed and the monolayers were covered with 2 ml culture medium containing 1% methylcellulose. After 7 days of incubation at 37°C, the cells were fixed and stained with a solution containing 0.1% (w/v) amido black, 12% acetic acid, 1.6% sodium acetate and 10% glycerol. After 30 min at room temperature, the overlay was removed, the cells were rinsed with aqua bidest and then air-dried. Plaque sizes (in mm^2^) were determined by measuring the x and y-axis of 15 (QM5 cells) or 20 (MDCK and VERO cells) randomly chosen plaques as described previously [40]. Differences in plaque size between maH7N7 and chH7N7 viruses were analyzed using the two-sample *t*-test. *P* values of < 0.05 were considered statistically significant.

### Virus replication kinetics

Growth rates of the viruses were determined after infection of MDCK or QM5 cells with viruses at a multiplicity of infection (m.o.i) of 0.001 TCID_50_ per cell. At 1 h p.i., the cells were washed once with PBS and 2 ml infection medium was added to the cells. Supernatants were collected after 8, 24, 32, 48, and 72 h of incubation at 37°C and titrated by end-point dilution in MDCK cells.

### Ethics statement

All animal experiments were authorized by the Animal Ethics Committee of the Animal Sciences Group, part of Wageningen UR (approval numbers: 2007053, 2007189, 2008009 and 2010032) and conducted in accordance with the Dutch law for animal experimentations.

## Competing interests

The authors declare that they have no competing interests.

## Authors’ contributions

RJ and LC conceived and designed the study, analyzed the data and wrote the paper. EV, EBL, SR and OL performed the laboratory work. NSZ participated in the ferret experiment and analyzed the pathological data. All authors read and approved the final manuscript.
